# The TGF-β Family in Glioblastoma

**DOI:** 10.3390/ijms25021067

**Published:** 2024-01-15

**Authors:** Irene Golán-Cancela, Laia Caja

**Affiliations:** Department of Medical Biochemistry and Microbiology, Biomedical Center, Uppsala University, SE-75123 Uppsala, Sweden; irene.golan@imbim.uu.se

**Keywords:** TGF-β, BMP, glioblastoma

## Abstract

Members of the transforming growth factor β (TGF-β) family have been implicated in the biology of several cancers. In this review, we focus on the role of TGFβ and bone morphogenetic protein (BMP) signaling in glioblastoma. Glioblastoma (GBM) is the most common malignant brain tumor in adults; it presents at a median age of 64 years, but can occur at any age, including childhood. Unfortunately, there is no cure, and even patients undergoing current treatments (surgical resection, radiotherapy, and chemotherapy) have a median survival of 15 months. There is a great need to identify new therapeutic targets to improve the treatment of GBM patients. TGF-βs signaling promotes tumorigenesis in glioblastoma, while BMPs suppress tumorigenic potential by inducing tumor cell differentiation. In this review, we discuss the actions of TGF-βs and BMPs on cancer cells as well as in the tumor microenvironment, and their use in potential therapeutic intervention.

## 1. Glioblastoma

Glioblastoma (GBM) is the most common primary central nervous system malignancy in adults; it has an incidence of 5 cases per 100,000 people per year. The median survival of GBM patients who have received standard care involving surgical resection followed by treatment with radiotherapy and temozolomide (TMZ) is 15 months [1,2,3]. In 2016, the World Health Organization (WHO) classified GBM according to the isocitrate dehydrogenase (*IDH*) mutation status, IDH-mutant (IDH-mt) or IDH-wild-type (IDH-wt) GBM; however, this classification has been reconsidered, as the IDH-mutant GBM aligns more closely with aggressive anaplastic astrocytomas; therefore, the Consortium to Inform Molecular and Practical Approaches to CNS Tumor Taxonomy (cIMPACT-NOW) has since proposed that the previously defined *IDH*-mt GBM now be referred to as astrocytoma, IDH-mt, grade 4 [4]. The current WHO 2021 classification defines glioblastoma as IDH-wt, and describes it as involving frequent alterations associated with mutations in the telomerase reverse transcriptase (*TERT*) promoter, amplifications of the epidermal growth factor receptor (*EGFR*), and single-copy loss of chromosome 10 and gain in chromosome 7 [5]. GBMs are characterized as invasive, vascularized, and necrotic, with high intratumoral heterogeneity. In 2010, they were classified into different molecular subtypes according to their transcriptomic profiles, proneural (PN), mesenchymal, and classical [6,7]. These subtypes can co-exist in the same tumor in different regions or at different times [7,8]. *PDGFRA* amplification can be found in all of the subtypes, but is most abundant in the PN subtype [9]; the mesenchymal subtype has a high *NF1* mutation burden, and the classical subtype displays high levels of *EGFR* amplification and few *TP53* mutations [4]. 

The cancer stem cell (CSC) hypothesis is the basis for many solid tumors, including brain tumors. It has been seen that a small group of glioma stem cells (GSCs) can exactly recapitulate the original tumor because of their significant clonogenicity and oncogenic potential. GSCs are also characterized by their high capacity for proliferation, self-renewal, and multilineage differentiation properties. They can grow as neurospheres in a serum-free medium and express several neuroprogenitor markers, like CD133, allowing them to perform cell sorting [10,11,12,13]. GSCs were identified in 2003 by isolating CD133+ GBM cells; these cells had higher proliferative capacity, self-renewal potential, and differentiation capability compared to CD133- GBM cells [13]. Glioblastoma cells become highly plastic in response to different growth factors and the microenvironment, shifting from a non-GSC state to a GSC state, this plasticity makes their identification difficult [14]. Several markers are currently used to identify GSCs: cell surface markers such as CD133, CD24, and CD44, and intracellular markers such as SOX4, Nanog, SOX2, MYC, BMI, differentiation inhibitor protein 1 (ID1), and nestin [15,16]. GSCs, similar to neural stem cells, respond to early embryogenic pathways that determine their cell fate and differentiation process; among these pathways are SHH, transforming growth factor β (TGF-β) family members, WNT ligands, NOTCH ligands, and Hippo signaling [17]. In this review, we focus on how different TGF-β family members play a key role not only in the fate of GSCs, but also in tumor microenvironment (TME).

## 2. TGF-β Signaling

TGF-β was isolated as a cytokine capable of inducing cellular transformation and anchorage-independent growth of fibroblast cell lines [18]. Since then, many proteins have been described as members of the TGF-β superfamily: TGF-β isoforms, bone morphogenetic proteins (BMPs), growth and differentiation factors (GDFs), activins, inhibins, nodal, and anti-Müllerian hormone (AMH) [19]. All of them can regulate cell proliferation, migration, differentiation, and apoptosis; therefore, it is known that they regulate a variety of events in embryonic development and normal physiology like tissue homeostasis. Perturbations in TGF-β signaling are detected in different diseases such as inflammatory conditions, connective tissue disorders, fibrosis, and cancer [19,20].

The TGF-β family includes 33 genes, each of which encodes a polypeptide characterized by containing a secretion signal peptide, a ~250-residue prodomain, and a ~110-residue growth factor domain. TGF-β is first synthesized as a latent and inactivated polypeptide that requires a functional activation to have biological activity. After dimerization followed by proteolytic cleavage, the result is a TGF-β molecule formed by two parts, a latency-associated peptide (LAP) and the mature TGF-β. The latent TGF-β is secreted bound to the latent TGF-β-binding protein (LTBP), and is deposited to the extracellular matrix (ECM) or associated with the plasma membrane of microglia cells. The activation of TGF-β consists of a sequential biochemical event that results in the release of the mature TGF-β from the LAP, and thus is able to bind to its specific receptor [21,22].

**Figure 1:**
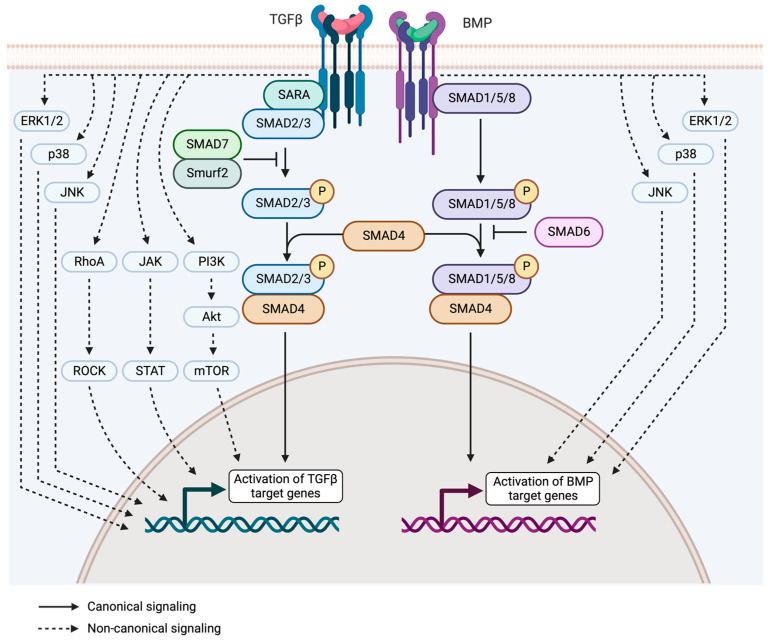
Transforming growth factor β (TGF-β) and bone morphogenetic proteins (BMP) signaling pathway. The canonical signaling pathway for TGF-β and BMP occurs through R-SMAD proteins, SMAD2/3 for TGF-β and SMAD1/5/8 for BMP. In both cases, Co-SMAD4 forms a complex with R-SMADs, and the timeric complex is translocated into the nucleus where it functions as a transcription factor. I-SMAD6/7 act as negative regulators of TGF-β and BMP signaling. The non-canonical signaling pathway works through non-SMAD proteins like ERK-MAP kinase, p38/JNK, RhoA/ROCK, JAK/STAT, or PI3K/AKT/mTOR. Created with BioRender.com.

The singular activities of TGF-β members (TGF-β1-3, Activin, and nodal) are determined by signaling through the type II (TβRII) and type I (TβRI) receptors (the activin receptor-like kinase, ALK4, 5, and 7), expressed in all cell types, as well as by multiple binding proteins and enzymes, that act as modulators of the signaling activity by adding a new level of signaling that takes place in the extracellular environment [21,22]. In the absence of the TGF-β ligand, TβRII and TβRI are present as homodimers at the cell surface. Both of them contain a cytoplasmatic kinase domain with serine/threonine kinase activity and tyrosine kinase activity [23,24]. TβRII is constitutively active. In the presence of the ligand, signaling is activated sequentially beginning with the binding of the ligand to TβRII and followed by the reclusion and phosphorylation of TβRI. Activated TβRI recruits and phosphorylates receptor-activated (R-) SMADs, SMAD2 and -3. These SMADs dissociate from TβRI and form a complex with the common (Co-) SMAD, SMAD4. This trimeric complex is translocated into the nucleus where it plays a role as a transcription factor for TGF-β target genes; it partners with other transcription factors to induce or repress gene expression. This pathway is the canonical signaling (Figure 1). The SMAD family also includes inhibitory I-SMADs, SMAD6/7, which negatively regulate the TGF-β signaling pathway by competing with R-SMADs to interact with TβRI, and thus prevent their activation [21,25,26,27]. SMAD7 also acts as a scaffold protein to recruit SMAD-specific E3 ubiquitin protein ligase 2 (SMURF2) to the TβR complex to facilitate its ubiquitination and subsequent degradation [21].

In addition to SMAD-dependent signaling, TβRs also work through different transducers, non-SMAD proteins like ERK-MAP kinase, p38/JNK, and the NF-κB pathway, PI3K/AKT/mTOR, JAK/STAT, or Rho-(like) GTPase pathway activation. These alternative pathways are collectively called non-SMAD signaling or non-canonical signaling (Figure 1), and have key roles in TGF-β regulation of apoptosis, epithelial–mesenchymal transition, migratory response, cell proliferation and differentiation, and matrix regulation [21,28,29].

BMPs are members of the TGF-β superfamily, which include BMPs and GDFs. Just as the TGF-β described above, BMPs bind to two different groups of serine/threonine kinase receptors, the type II and type I receptors. Among the type II receptors, BMPs bind to BMP type II receptor (BMPR-II), activin type II receptor (ActR-II), and activin type IIB receptor (ActR-IIB); type II receptors are constitutively active. The type I receptors are ALK1, 2, 3, and 6. Upon BMPs binding to type I and type II receptors, type II receptors phosphorylate and activate type I receptors. Then, type I receptors phosphorylate the R-SMADs, SMAD1, SMAD5 and SMAD8, which form a heteromeric complex with the Co-SMAD, SMAD4, and translocate to the nucleus where they bind other transcription factors to induce or repress the expression of BMP target genes. Similar to TGF-β signaling, the I-SMADs SMAD6 and SMAD7 repress BMP signaling (Figure 1). BMPs also lead to the activation of non-SMAD signaling pathways, such as the ERK-MAPK, p38/JNK, and PI3K pathways [30,31]. Several extracellular BMP antagonists can bind to BMPs and reduce their availability to interact with the BMP receptors; these antagonists include noggin, chordin, gremlin, and others [32].

## 3. TGF-β Family and Cancer in General

### 3.1. TGF-β Effects in Cancer

There is a term known as “TGF-β paradox” that refers to the dual effect of this cytokine on cancer development. TGF-β can act as a potent tumor suppressor in early-stage tumors by inducing potent antiproliferative responses, cell differentiation, and apoptosis; however, in the late stage of cancer progression, TGF-β can promote many of the hallmarks of cancer like plasticity via epithelial-to-mesenchymal transition (EMT), evasion of the immunosuppressive environment, and induction of angiogenesis and systemic dissemination [27,33,34].

A lot of cancer types show high concentrations of TGF-β in the tumor microenvironment, TME, and many studies support the fact that TGF-β has an important role in TME. The source of this cytokine is both from tumor cells and normal tumor-adjacent cells like fibroblasts, endothelial and immune cells, mesenchymal cells, and adipocytes. The biological role of TGF-β during tumor progression not only depends on its signaling in malignant epithelial cells, but also in these tumor-adjacent cells. It has been described that TGF-β promotes the mobilization of mesenchymal precursors and generation of myofibroblasts, as well as the recruitment of fibroblasts. Furthermore, TGF-β can induce transdifferentiation of endothelial cells into mesenchymal cells, and the tumor fibroblasts may originate from TGF-β-induced EMT [34].

TGF-β regulates the expressions of EMT transcription factors (SNAI1/2, TWIST, ZEB1/2), as well as miRNAs, lncRNAs, and mRNA translational mechanisms, to induce the EMT program: expression of ECM genes, re-structuring of cell contacts and actin-based cytoskeleton, as well as the expressions of many other growth factors and cytokines (FGF, PDGF) [35]. Recently, new studies propose a regulation of TGF-β-induced EMT through the TGF-β/SMAD/RREB1 axis (a RAS transcriptional effector) [36] or TNF-α [37].

Concerning immune evasion, TGF-β is involved in suppressive and inflammatory immune responses, and changes immunity under different conditions [38]. An increase in TGF-β levels in TME pushes the switch from naive CD4+ T cells to Treg cells at the expense of its differentiation to effector T cells, and it blocks the function of dendritic cells, Th1 cells, and natural killer (NK) cells. In NK cells, it has been described that TGF-β inhibits mTOR-dependent metabolic activity, recapitulating the effect of mTOR inhibition by rapamycin, an immunosuppressant pharmacological agent used in clinics to inhibit rejection in organ transplantation [39]. TGF-β also promotes the acquisition of an antitumorigenic M2 and N2 phenotype in tumor-associated macrophages (TAMs) and tumor-associated neutrophils (TANs), respectively. All of these modifications induce immune suppression in the TME during tumor progression [40].

TGF-β induces metastases to bone, liver, lung, and other tissues of specific cancer types, such as breast, lung, gastric, and prostate cancers [27,41]. TGF-β promotes migration and invasion of cancer through its SMAD-dependent signaling pathway. In prostate cancer cells, the burden is on SMAD7, which is described as a negative regulator of TGF-β signaling; however, it has also been shown that it can promote the transcription of c-Jun and HDAC6 in the nucleus, two genes related to tumor invasion [42]. It has been proven that TGF-β by itself can promote invasiveness in cancer, but in some cases, like in breast cancer cells, it has been shown to collaborate between canonical TGF-β/SMAD3 signaling and the EGF/EGFR pathway to facilitate cancerous migration and invasive abilities [43]. Moreover, after the demonstration that TGF-β can control the exosome protein content in lung adenocarcinoma [44], it has been reported that the TGF-β-mediated exosomes have a high Lnc-MMP2-2 content, and that this noncoding RNA regulates lung cancer invasion and vascular permeability through the expression of MMP2 [45]; that, together with other matrix metalloproteinases, digests the extracellular matrix, facilitating the infiltration of malignant cells.

### 3.2. BMPs Effects in Cancer

Similar to TGF-β, BMPs regulate proliferation, survival, invasion, and self-renewal capacity in different cancer types. They have been described to act as both tumor suppressors and oncogenes. 

In hepatocellular carcinoma (HCC), BMP4 promotes cell cycle progression by upregulating the expression of cyclin-dependent kinase (CDK)1 and cyclin B1 in HCC cells, while in diffuse-type gastric carcinoma cells, BMP4 induces G1 arrest by upregulating the expression of *p21* cyclin kinase inhibitor via the SMAD pathway [30]. BMP2 in breast cancer cells also induces cell cycle arrest by increasing *p21* expression [46]. Among the tumor suppressor roles of BMPs, they have been reported to induce cancer stem cell differentiation in colorectal CSCs by BMP4 [47] and in GSCs by BMP4 and BMP7 [48,49]. In prostate cancer, BMP7 upregulates the expression of *NDRG1*, a metastasis suppressor gene, which induces senescence in CSCs, leading to tumor dormancy [50].

BMPs induce EMT in different cancer types. BMP2 upregulates STAT3 levels to promote EMT, migration, and invasion in colon cancer cells [51]. In ovarian cancer cells, BMP2 promotes cell proliferation and self-renewal capacity via c-Kit induction as well as cell migration and invasion via EMT [52]; these effects are TNFRSF12A/FN14-dependent, as silencing of *FN14* suppresses BMP2 actions in ovarian cancer cells [53]. In gastric cancer cells, BMP2 activates the PI-3 kinase/Akt pathway to promote invasion [54]. BMP4 induces EMT in breast cancers via Notch signaling; it promotes invasion in breast cancer cells and colon cancer cells [55]. In squamous cell carcinoma, BMP4 not only induces EMT, but also increases the expressions of stem cell markers *CD44*, *BMI*-1, and *ABCG2* [55]. However, BMP6 was found to inhibit EMT in breast cancer cells by repressing the expressions of metalloproteases *MMP1* and *MMP9*, leading to the inhibition of invasion and metastasis [55].

In summary, TGF-β family members act as tumor suppressors or promoters, depending on the cell type and other factors in the tumor microenvironment that can counteract their actions.

## 4. TGF-β Family Effects in Glioblastoma

### 4.1. TGF-β Effects in GBM

As we mentioned above, in advanced stages of tumors, including glioblastoma, the TGF-β pathway acts as an oncogenic factor. A high concentration of this cytokine is found in malignant gliomas and correlates with a poor prognosis [56]. Malignant gliomas differentially express the three TGF-β isotypes, TGF-β_1_, TGF-β_2_, and TGF-β_3_. The in vivo mRNA expression levels of TGF-β_1_ and TGF-β_2_ are higher than TGF-β_3_ levels, and the expression of any of the isotypes correlates with poor survival in glioblastoma [57,58,59]. The TGF-β_2_ concentration is high due to an autocrine loop by which TGF-β_1_ cooperates with PI3K/AKT and p90RSK to induce TGF-β_2_ expression through CREB1 [58]. Interestingly, inhibiting TGF-β_3_ negatively regulates the SMAD-dependent signaling pathway, even in the presence of TGF-β_1_ and TGF-β_2_ [59]. TGF-β activity in GBM is modulated by several deubiquitinating enzymes (DUBs); first, it was described that TGF-β activity could be enhanced due to the amplification of a deubiquitinating enzyme, *USP15*, which binds to the SMAD7–SMURF2 complex and stabilizes type I TGF-β receptors, resulting in enhanced TGF-β signaling. GBM patients with a higher copy number of *USP15* have poorer survival [60]. More recently, it was published that loss in *USP2* expression due to DNMR3A-mediated aberrant DNA methylation enhances TGF-β signaling and GBM progression. USP2 leads to de-ubiquitination and stabilization of SMAD7, and *USP2* loss results in an increased SMAD7 ubiquitination and increased phospho-SMAD2 [61]. E3 ubiquitin protein ligase 2 SMURF2 activity is activated by phosphorylation at Thr^249^. Interestingly, the level of SMURF2 Thr^249^ phosphorylation is lower in GSCs compared to differentiated glioma cells, leading to increased TGF-β activity; SMURF2 Thr^249^ phosphorylation regulates GBM tumor growth, invasiveness, and self-renewal of GSCs [62]. Interestingly, glioblastoma patients with low expression levels of *USP26* have a worse prognosis, which correlates with in vitro experiments where knock-down of *USP26* leads to increased phospho-SMAD2, and cell migration [63]; since USP26 de-ubiquitinates and stabilizes SMAD7, this leads to downregulation of TGF-β activity. Finally, glioblastoma patients express high levels of USP4, which in turn can enhance the expression of TβRI, resulting in increased phospho-SMAD2 [64].

GBM cells are resistant to TGF-β tumor suppressor effects. One explanation is the high levels of the transcription factor FoxG1 expressed in GBM cells, which interacts with FoxO3, inhibiting the capacity of TGF-β to induce *p21Cip1* expression [65], responsible for the inhibition of the activity of cyclin-dependent kinase (CDK)2 and CDK4 complexes and promoting cell cycle G1 phase arrest [66]. In contrast, TGF-β promotes tumorigenesis in GBM by inducing proliferation, invasion, and self-renewal. High levels of phospho-SMAD2 correlate with high levels of proliferating cells in GBM patients; to promote proliferation, TGF-β induces *PDGFBB* expression in a SMAD2/3-dependent manner [56]. The transcription factor Olig1 is also involved in the transcriptional regulation of *PDGFBB* by SMAD2/3 [67]. TGF-β induces the expression of another TGF-β family member, nodal, to promote growth of glioma cells and inhibit apoptosis [68].

TGF-β is known to induce migration and invasion in several cancer cell types [69], including glioblastoma. It has been shown that TβRII knock-down impairs TGF-β-induced migration, invasion, and tumor growth in nude mice [70]. TGF-β promotes glioma cell invasion via multiple pathways: increasing the expression of *lncRNA-ATB*, which activates both signaling pathways NF-κB and p38/MAPK to promote cell invasion [71], or through the IFITM3/STAT3 axis [72]. Moreover, TGF-β downregulates the lncRNA *LINC00707* to favor glioblastoma cell invasion, as LINC00707 interacts with SMAD proteins to limit TGF-β signaling [73]. Not only has TGF-β_1_ been shown to promote invasion in GBM, but the silencing of TGF-β_3_ inhibits GBM cells’ capacity for invasiveness [59]. TGF-β induces the expression of thrombospondin 1 (*TSP1*) through SMAD activation to enhance the formation of microtube networks in GBM, a cytoplasmic extension that is important for cell communication, invasion, and treatment resistance [74].

TGF-β promotes the expression of mesenchymal markers in GBM cells via ZEB1 and SMAD2 enhancing the invasive phenotype of GBM cells [75]. Recent studies have identified a relationship between insulin-like growth factor (IGF)-1 and TGF-β signaling through miRNA-4286 to regulate EMT and cell invasion in GBM [76]. The *LINC00645* is induced by TGF-β and plays a key role in TGF-β-induced mesenchymal differentiation by sponging miR-205-3p, which in turn targets ZEB1, thus promoting ZEB1 expression as well as TGF-β-induced glioma cell invasion and migration [77]. On the other hand, TGF-β also increases the expression of lncRNA *UCA1*, which targets miR-1 and miR-203, resulting in an increase in Slug expression that leads to EMT and stemness [78]. TGF-β not only regulates lncRNA to promote mesenchymal differentiation and invasion, but also miRNAs such as inducing the expression of mirR10b; this promotes EMT, lamellipodia formation, and invasion [79]. TGF-β upregulates the expression of Claudin 4 and its translocation to the nucleus to activate the NF-κB/TNFα signaling pathway to induce mesenchymal transformation of GBM cells and enhance cell invasion and tumor growth [80]. 

TGF-β_1_ has a function in cancer metabolic reprogramming [81,82]. Cancer cells depend on aerobic glycolysis rather than oxidative phosphorylation for energy production. GBM tumors can use glucose for both glycolysis and oxidative phosphorylation [83]. In glioblastoma cells, TGF-β_1_ induces the expression of PFKFB3, an enzyme that controls the conversion of fructose-6-phosphate to fructose-2,6-bisphosphate, which is important for the dynamic regulation of glycolytic flux as well as the expression of the glucose transporter *GLUT1* [84,85]; NOX4-derived reactive oxygen species mediate TGF-β_1_ induction of *GLUT1* expression [85,86].

TGF-β has been reported to induce resistance to chemotherapy, target therapy, and immunotherapy [87]. In GBM, TGF-β contributes to TMZ resistance by increasing MGMT accumulation and repressing miR-198 levels [88]. TMZ treatment enhances connective tissue growth factor to mediate TMZ resistance through activation of TGF-β signaling pathways [89].

TGF-β has a role in the maintenance of GSCs. It was initially described that TGF-β induces the expression of *LIF* in a SMAD2/3-dependent manner, which in turn activates the JAK/STAT pathway; this results in the induction of GSC self-renewal and blocking their differentiation, thereby promoting oncogenesis [11]. Later, we described how TGF-β induction of *LIF* expression was NOX4-dependent, and how NOX4-induced reactive oxygen species after TGF-β treatment are required for TGF-β-induced GSCs proliferation and self-renewal [85]. TGF-β induces the expression of *SOX4*, which in turn enhances the expression of the stem cell transcription factor *SOX2* [90]. Moreover, TGF-β increases the number of GSCs that are positive for CD44+ and ID1 [91]; inhibition of the TGF-β receptor results in downregulation of *SOX2* and *SOX4* expression, as well as a decrease in the GCS subpopulation of CD44^high^/Id1^high^, resulting in decreased oncogenic potential [91].

### 4.2. BMPs Effects in GBM

BMPs have been described to regulate the telencephalic neural progenitors’ differentiation state from neuronal fate to astrocytic fate by upregulating glial fibrillary acidic protein (GFAP) expression [92]. In adult-derived hippocampal progenitor cells, BMP2 and BMP6 via ALK2 and ALK6 induce astrocytic differentiation while blocking oligodendrocyte marker expression [93]. Later on, it was published that BMP4 impairs the tumor-initiating capacity of GSCs by reducing cell proliferation and increasing the expressions of differentiation markers of astrocytic, neuronal, and oligodendrocyte lineage [48]. This indicates that BMPs have an opposite role to TGF-β_s_ in GSCs. In agreement with Piccirillo et al. [48], several research groups have reported the role of BMPs as a tumor suppressor for GBM. There is a subset of GSCs in which the BMP receptor 1B (*BMPR1B*) is epigenetically silenced by EZH2; in such cells, an overexpression of BMPR1B restores GSCs’ differentiation capacity and reduces their tumorigenicity [94]. We described that BMP7 promotes astrocytic differentiation of glioma-initiating cells by inducing the expression of the EMT transcription factor *SNAIL* in a SMAD1/5-dependent manner; the induction of Snail is required for BMP7 to be able to induce the expression of GFAP, an astrocytic marker [49]. Moreover, Snail can repress the expression of *TGFB1* by interacting with SMAD proteins [95]. BMPs affect and promote the methylation of the *PROM1* gene promoter, a gene that encodes CD133, thus diminishing their stem cell properties. BMPs induce the expression of paired related homeobox 1 (*PRRX1*); its isoform pmx-1b interacts with the DNA methyltransferase 3A, DNMT3A, responsible for the methylation and epigenetic changes induced by BMP7 [96]. BMP9 has also been shown to act as a tumor suppressor in GBM by inhibiting proliferation, invasion, and inducing GSCs differentiation as well as counteracting GSCs’ trans-differentiation ability towards tumor-derived endothelial cells [97]. 

BMP4 not only induces GSCs differentiation, but also induces growth arrest in GBM cells [98]. This effect is observed in a subset of GBM cells from the proneural subtype, where BMP4 can downregulate *SOX2* expression, but not completely, as low levels of *SOX2* are probably required in BMP4-sensitive GBM cells [99]. Interestingly, single-cell RNA-Seq has shown that higher levels of *OLIG1/2* expression predict that those GBM cells will respond to BMP4-induced cell arrest [100]. Moreover, BMP2, 4, and 7 also induce GSCs apoptosis via two different mechanisms: (1) induction of distal-less homeobox 2 (*DLX2*), which is necessary for BMP4 and BMP7-induced apoptosis and sensitization to the anti-epileptic-compound valproic acid [101]; (2) induction of EPHA6 phosphorylation, which enhances BMP2-induced apoptosis [102].

TGF-β has been shown to increase the number of GSCs that are positive for CD44+ and ID1 [91]. Interestingly, ID1 suppresses *BMPR2* expression through mir-17 and miR-20a in a MYC-dependent manner to support GSCs’ intrinsic stemness [103]. Another mechanism that GSCs use to maintain their stemness is to secrete gremlin, a BMP antagonist, to counteract the higher levels of endogenous BMPs found in more differentiated GBM cells [104].

BMP2 was described to sensitize resistant GBM cells to TMZ-induced cell death; and co-treatment with BMP2 and TMZ was described to induce differentiation, downregulation of HIF1α activity, and consequently diminishing the expression of MGMT [105], which mediates TMZ resistance. However, other reports show that BMP4 confers resistance to TMZ by upregulating *p21* expression, also necessary for BMP4 to inhibit GBM cell proliferation [106].

## 5. TGF-β Family in Glioblastoma Microenvironment

Initially, GBM has been characterized by its histological features and genetic alterations; however, after years of studies, more and more importance is linked to the role of the tumor microenvironment in relapse and therapeutic resistance [107,108,109].

During the first steps of glioma development, competent immune cells, like microglia, natural killer (NK) cells, macrophages, and dendritic cells (DCs), try to impair tumor growth through the release of cytotoxic and proinflammatory factors TNF-α and IL-6, which recruit T CD4^+^ helper and T CD8^+^ cytotoxic cells from the periphery to the tumor. Interleukin (IL)-1β also helps in blood–brain barrier destruction, which favors the infiltration of myeloid-derived suppressor cells (MDSCs) and macrophages [110]. Tumor-infiltrated leukocytes constitute 40% of the tumor mass; among them, the main populations are tumor-associated macrophages (TAM) and microglia. There is cross-talk between TAMs and MDSCs and surrounding tumor cells that favors the immunosuppression: tumor cells secrete macrophage migration inhibitory factor that promotes the recruitment of MDSCs, macrophages, and microglia. Tumor cells also secrete factors MCP1, CSF1, and MCP3, which promote the transition from the proinflammatory M1 state to the anti-inflammatory M2 phenotype [110]. Recruited macrophages start to lose their phagocytic ability, cytotoxic T cells stop proliferating, and the amount of regulatory T cells becomes higher in the tumor site, altogether generating chronic immunosuppression and tumor tolerance [110].

Microglia have been described to stimulate invasiveness in glioblastoma cells [111,112]; this effect is dependent on their ability to release TGF-β, and is blocked by silencing TβRII in glioblastoma cells or using anti-TβRII antibodies [70]. Microglia and TAMs release TGF-β that promotes *MMP9* expression in GSCs, increasing their invasiveness [113]. TGF-β1 may be responsible for M2-like macrophage polarization and inhibition of TGF-β-R1/TGF-β-R2, which decreases the expression of M2-like polarization genes in macrophages [114]. On the other hand, it has been described that in turn, M2 secretes TGF-β1 that activates the SMAD2/3 pathway, promoting stemness via SOX4 and SOX2 upregulation and cell migration via EMT program activation [115].

**Figure 2:**
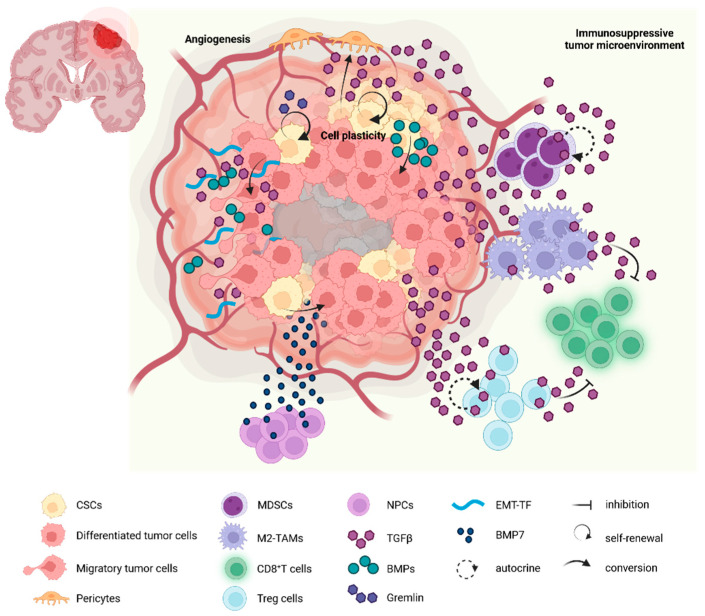
The roles of TGF-β and BMP in the glioblastoma microenvironment. Tumor-infiltrating immune cells contribute to the immunosuppressive microenvironment through the communication established between them and the tumor cells via TGF-β. TGF-β also supports cell plasticity by helping the differentiation of cancer stem cells (CSCs) to pericytes, and self-renewal of CSCs. On the other hand, BMPs contribute to cell differentiation, from CSCs to differentiated tumor cells. Both TGF-β and BMPs, together with epithelial to mesenchymal transcription factors (EMT-TF), have important roles in the acquisition of migratory characteristics by tumor cells. Created with BioRender.com.

TGF-β has been reported to enhance the recruitment of regulatory T cells (Treg) in gliomas (Figure 2); interestingly, treatment with an anti-TGF-β antibody, m1D11, diminishes the infiltration of Treg in these tumors [116]. Moreover, IL17^+^ Tregs suppress CD8^+^ T cell proliferation in a TGF-β-dependent manner [117]. T-reg also supports tumorigenesis by enriching GSC populations by secreting TGF-β, which in turn promotes tumor cells to secrete IL6 that induces stemness in glioblastoma cells [118]. TGF-β has been shown to downregulate the expression of the activating receptor *NKG2D* in CD8^+^ T cells and NK cells; silencing of TGF-β in glioma cells promotes their recognition by CD8^+^ T cells and NK cells [119,120].

The GBM microenvironment is not only associated with changes in the immune system, but also with aberrant neovascularization and dynamic ECM alterations; TGF-β has a role in each of these processes. TGF-β is related to immunosuppression through the inhibition of T cell activation and proliferation and also NK cell activity, the blocking of IL2 production, and the stimulation of Treg activity. However, this cytokine can also promote tumor growth (by sustaining GSC), angiogenesis, and invasion (through the upregulation of molecules like MMP-2) [121]. TGF-β released by GBM cells acts on endothelial cells and promotes angiogenesis by increasing the expression of insulin-like growth factor-binding protein 7 (IGFBP7) [122]. TGF-β reduces the expressions of the adhesion proteins intercellular adhesion molecule 1 (ICAM1) and vascular cell adhesion molecule 1 (VCAM1) in endothelial cells, which results in less T cell transmigration [123].

Neural precursor cells can also interact with GBM cells by secreting BMP7, which, as described above, induces GSCs differentiation [124]. A BMP7 variant, BMP7v, is also able to reduce angiogenesis in GBM xenografts [125]. In general, BMPs play a tumor suppressor role by acting on both tumor cells and the tumor microenvironment, while TGF-β has tumor-promoting effects in GBM.

## 6. Therapeutic Perspectives

The standard approach to therapy for newly diagnosed GBM is maximal safe surgical resection followed by concurrent radiotherapy with temozolomide and further adjuvant temozolomide. Together with adjuvant temozolomide, the patient can also receive low-intensity alternating electric fields. Despite this, GBM easily recurs and results in sad outcomes because there is no standard of care in these cases. There are only potential options such as surgical resection, reirradiation, systemic therapies with lomustine or bevacizumab, combined approaches, or supportive care, all of which depend on the circumstances of the patient [126].

### 6.1. Current Approaches to Disrupt TGF-β Signaling

Several pharmacological interventions aim to target specific mediators of the TGF-β signaling pathway. In this section, we provide a short overview of recent advancements. 

Two different neutralizing antibodies have been tested in clinical trials. LY3022859 is an anti-TβRII IgG1 monoclonal antibody that blocks TGFβ binding to TβRII; in mice models, it shows antitumor effects [127]. However, when tested in advanced solid tumors in a phase 1 clinical trial, the patients suffered from uncontrolled cytokine release, and the treatment was considered unsafe [128]. The more promising neutralizing antibody is fresolimumab (GC1008), a human IgG4κ monoclonal antibody that neutralizes TGFβ1, 2, and 3. It has already passed phase 1 clinical trials for patients with malignant melanoma or renal carcinoma, where it showed acceptable safety and antitumor activity [129]. There have been several phase 2 clinical trials that have shown good tolerance of this antibody in patients with glioma, metastatic breast cancer, or relapsed malignant pleural mesothelioma [127,130,131]. In 2023, a phase 2 clinical trial of this antibody in early-stage non-small cell lung cancer (NSCLC) was concluded (NCT02581787), but no results have yet been published.

Integrins are major activators of TGFβ ligand, including αvβ1, αvβ3, αvβ5, αvβ6, and αvβ8 [132]. Interestingly, αvβ6-neutralizing antibody 264RAD has been reported to suppress TGFβ signaling and reduce tumor growth in an αvβ6-positive human pancreatic ductal adenocarcinoma (PDAC) xenografts mice model [133].

TGFβ ligand traps are chimeric fusion proteins based on the TGFβ receptor ectodomain that are designed to prevent TGFβs from binding to their receptors. AVID200 is a selective trap of TGFβ1 and TGFβ3 that has shown antitumor efficacy in breast cancer models in mice (4T1 cells) [134]. Moreover, a phase I clinical trial has proven AVID200 to be safe and well tolerated by patients [135].

There are several small-molecule kinase inhibitors of TβR kinases, and some of them are currently in clinical trials. Galunisertib (LY2157299) is an orally available small-molecule inhibitor that selectively binds to TβRI (and weakly to TβRII) and inhibits its kinase activity. Preclinical studies showed promising antitumor growth results. Initial phase I clinical trials showed acceptable safety and dose tolerance for patients with advanced solid tumors [132]. A phase 2 study in advanced hepatocellular carcinoma showed prolonged overall survival when combining galunisertib and sorafenib. However, in a phase 2 study of patients with recurrent glioblastoma, this inhibitor failed to improve overall survival when combined with lomustine [136]. Another orally available small-molecule inhibitor of TβRI, vactosertib (TEW-7197), has been tested in phase 1 clinical trials for patients with advanced solid tumors, and has shown a favorable safety profile [137]. Its antitumor efficacy is currently being investigated in phase 2 clinical trials in patients with metastatic gastric adenocarcinoma [138], among other solid tumors (such NSCLC, osteosarcoma, and colorectal cancer). Another potent ATP-competitive TβRI inhibitor is LY3200882, which has shown a tolerable safety profile and early signs of antitumor efficacy for patients with advanced pancreatic cancer [139]. There are currently two different phase 2 clinical trials testing LY3200882 in advanced cancer (NCT04158700) and colorectal cancer (NCT04031872).

Antisense oligonucleotides are short oligonucleotides that suppress the expressions of specific genes by blocking their translation. Trabedersen (AP12009) is an antisense oligodeoxynucleotide that specifically targets TGFβ2 mRNA. Trabedersen showed good safety results in phase 1 clinical trials; phase 2 clinical trials in glioblastoma or anaplastic astrocytoma patients allowed determination of the optimal dose. However, no phase 3 clinical trials have been conducted [132].

More strategies are appearing based on directing therapy toward the cancer microenvironment. In breast cancer, it has been shown that blocking TGF-β signaling in CD4+ T cells via a bispecific receptor that attaches the TGF-β-neutralizing TβRII extracellular domain to ibalizumab (a non-immunosuppressive CD4 antibody) causes a remodulation of the tumor microenvironment, and suppresses tumor growth [140]. Some therapies in which anti-TGFβ and immunotherapy are combined have entered clinical evaluations with patients, with some examples described here. PM8001 is a bifunctional protein composed of the extracellular domain of the TGF-β RII receptor (acts as a TGF-β “trap”) fused to a humanized anti-PD-L1 IgG1 single-domain antibody. PM8001 showed an acceptable safety profile and promising antitumor activity in advanced solid tumors in phase 1 clinical trials [141], and phase 2 studies are currently ongoing (ChiCTR2000033828). HCW9218 is a heterodimeric bifunctional fusion protein complex that comprises IL15 immunostimulatory and TGF-β antagonistic activities. HCW9218 can neutralize TGF-β-mediated immunosuppression, as well as promote proliferative, metabolic, and tumor-targeted cytotoxic activities of NK cells and CD8^+^ T cells [142]. Currently, there are two clinical trials, phase 1 and phase 1b/2, which are recruiting patients with advanced solid tumors (NCT05322408) and advanced pancreatic cancer (NCT05304936). AK130 is a humanized Fc-mutant anti-TIGIT antibody fused with TGFβ-RII protein. AK130 combines an antibody against TIGIT, T-cell immunoglobulin, and ITIM domain, and is expressed on T and NK cells that can interact with its ligands (CD155 and CD122), resulting in inhibitory signaling in T cells and promoting exhaustion of lymphocytes. AK130 has shown promising results in hepatocellular carcinoma mice models [143]. AK130 will be tested for its safety, tolerability, pharmacokinetics, and antitumor activity in phase 1 clinical trials in patients with advanced malignant tumors (NCT05653284). M7824 is bintrafusp alfa, a first-in-class bifunctional fusion protein targeting TGF-β and programmed death ligand 1 (PD-L1). In comparison with monotherapies, some preclinical studies in mouse models have shown efficacy in the use of molecules that target both PD-L1 and TGF-β. M7824 suppresses tumor growth, metastasis, extends survival, and confers long-term protective antitumor immunity (evaluated in multiple tumor types) [144,145]. M7824 has shown a favorable safety profile and clinical efficacy in phase 1 clinical trials, including patients with advanced solid tumors, NSCLC, recurrent GBM, cervical cancer, metastatic TNBC, heavily pretreated CRC, or human papillomavirus (HPV)-associated cancers [132]. M7824 is now in phase 3 clinical trials for biliary tract cancer (NCT04066491) and NSCLCs (NCT03631706).

### 6.2. TGF-β Targeting in GBM

The major therapeutic complication is the ineffective delivery of drugs to tumors. Moreover, in regards to GBM, the presence of the blood–brain barrier (BBB) and the unique tumor and immune microenvironment are obstacles to be considered in the design of new therapies. For a long time, the brain was considered to be an immune-privileged organ because of restricted immune cells traffic into the CNS through the BBB [146], generating a unique immune microenvironment. However, in a malignant brain tumor, the BBB can be destroyed and the permeability of immune cells increases [147]. Under this situation, immune surveillance in the CNS becomes quite complex.

Many research projects focus on solving this problem and proposing new therapeutic targets, including molecules related to TGF-β (Table 1). For example, the option of using TGF-β to enhance the homing capacity of human adipose-derived stem cells (hAMSCs) to GBM and using them as a drug delivery vehicle showed enhanced therapeutic efficacy by an increased number of migrated hAMSCs to target sites, decreased tumor volume, and prolonged survival time in a murine model of GBM [148]. Another useful therapeutic delivery system is hematopoietic stem cell (HSC)-derived myeloid cells, since they also home efficiently to GBM. HSC gene therapy is successfully being used in clinics to treat non-cancerous brain disorders, and a preclinical GBM mouse model based on TGF-β-blocking HSC gene therapy in combination with irradiation has been shown to reduce tumor burden and significantly prolong survival compared with monotherapies and control groups [149].

As we mentioned above, TMZ is the medication used to treat GBM. A study has demonstrated that a conjugate of temozolomide and perillyl alcohol, NEO212, blocks TGF-β and Notch pathways; consequently, it arrests endothelial-to-mesenchymal transition (EndMT) in vivo. Invasiveness and pro-angiogenesis properties associated with tumor-associated brain endothelial cells (BEC) are reduced after treatment with NEO212 [150]. Other molecules that have been shown to be potent inhibitors of TGF-β in GBM are cholesterol-lowering statins, which could be used as adjunct therapy in GBM treatment, as simvastatin reduced invasion at the tumor margin and prolonged mouse survival [151].

Cancer immunotherapy was developed from the concept of immunosurveillance, by which the immune cells are capable of detecting and destroying tumor cells. However, the process is not simple because the immune system may not be strong enough to kill cancer cells. Cancer cells produce signals that stop the immune system from attacking it, and/or cancer cells can also acquire the capacity to avoid the immune system through a process called immunoediting [152]. These are the barriers that immunotherapy must overcome to break immunoresistance and destroy the tumor [153]. GBM has been shown to display a variety of tricks to evade the immune system and be highly immunosuppressive; most of them are based on manipulating the microenvironment and stimulating healthy cells to participate in tumor growth [154]. Among all of these cells, macrophages and microglia are the most common cells in brain tumors, comprising 30–50% of the tumor mass. There is communication between glioma cells and macrophages through TGF-β: glioma cells secrete TGF-β that participates in M2 polarization by macrophages [155]; in turn, macrophages secrete this immunosuppressive cytokine, leading to immune surveillance suppression and promotion of tumor angiogenesis, invasion, and metastasis [156]. Another important player here is the integrin α_v_β_3_, which participates in endothelial cell–macrophage interaction and regulates inflammation-derived angiogenesis. The dual inhibition of α_v_β_3_ integrin and TβRI in the GBM microenvironment suppresses endothelial cell proliferation and M2 macrophage polarization, which is an interesting adjuvant therapeutic target in GBM treatment [114].

Recently, an alternative strategy has emerged that uses inhibitors of the αv integrin/TGF-β axis in GSCs as a combined approach with NK cell immunotherapy. This approach is based on the fact that infiltrated NK cells within the tumor make contact, via CD9 and CD103, with αv integrin on GSCs; this cell contact mediates the release of TGF-β1 by GSCs, which in turn suppresses the cytotoxic function of NK cells [157]. Thus, with NK cell immunotherapy, the patient’s NK cells are replaced with healthy NK cells that will conserve their cytotoxic function; this, in combination with galunisertib treatment, led to the best overall survival in mice. Another approach tested by the authors was the use of *TGFBR2*-KO NK cells, which showed even better results than the NK WT cells injected in combination with galunisertib. Currently, there is a phase 1 clinical trial that is currently recruiting patients, in order to determine the best dose as well as the side effects of engineered natural killer (NK) cells *TGFBR2*-KO and NR3C1 (cord blood [CB]-NK-TGF-betaR2-/NR3C1-) in treating patients with recurrent glioblastoma (NCT04991870).

As mentioned above, there are currently no established treatments for relapse or recurrent GBM. Some projects use recurrent or therapeutic resistance GBM models to test potentially effective molecules for treatment. One of these molecules is disulfiram (DSF), which has been shown to sensitize resistant GBM to galunisertib in an orthotopic xenograft GBM model [158]. An alternative approach against recurrent GBM is the use of the oncolytic herpes simplex virus (oHSV), which has been largely studied in preclinical and clinical trials with promising therapeutic effects on GBM. In an orthotopic recurrent GBM mouse model, systemic treatment with TβR inhibitor has been shown to reinforce the antitumor effects of single intratumoral oHSV injections, resulting in a 60% cure rate in tumor-bearing mice [159].

There are currently few published clinical trials based on the treatment of GBM by affecting the TGF-β signaling pathway. Two of them combine the TGF-β2 inhibitor, AP12009, with temozolomide or PCV to treat recurrent or refractory high-grade glioma (NCT00431561 completed); the other involves temozolomide and a drug delivery system to treat recurrent or refractory anaplastic astrocytoma or secondary GBM (NCT00761280 terminated). Another two studies use the TβRI inhibitor, LY2157299 (galunisertib): one combines it with lomustine to treat recurrent GBM (NCT01582269, active but not recruiting), and the other combines it with radiation and temozolomide in patients with newly diagnosed malignant glioma. This latest study, NCT01220271, has been completed, and has a publication concluding that the rate of disease control was higher in treatment with galunisertib plus radiotherapy compared to radiotherapy alone; however, no change was found in the probability of survival [160]. The neutralizing antibody, fresolimumab, has also been tested in a phase 2 clinical trial for glioma and glioblastoma, where it has been shown to penetrate recurrent high-grade gliomas very well but did not result in clinical benefit (NCT01472731) [131].

A phase 1 clinical trial with M7824, a bifunctional fusion protein targeting TGF-β and PD-L1, in an expansion cohort of 35 patients with recurrent GBM, progressed after radiotherapy plus temozolomide. The preliminary efficacy of this phase I study showed that 8 patients out of 35 (22.9%) treated with M7824 exhibited disease control with the safety profile being feasible [161].

The ability of BMPs to promote differentiation [48,49] has led to explorations of the use of BMPs as therapeutic agents. For example, it has been reported how BMP4-engineered vaccinia viral particles induce inhibition of GSC growth, decrease their stem cell properties, and promote differentiation in vitro. Then, the BMP4-producing vaccinia viruses were delivered intracranially in an orthotopic mouse model of GBM, and resulted in tumor regression and better mice survival [162]. Interestingly, the use of a biodegradable device that allows for a controlled release of bioactive BMP7 in vitro has been used, where BMP7 was encapsulated in a heparin core and surrounded by a biodegradable polyester matrix shield. This nano-system could release bioactive BMP7 in a controlled manner for 2 months, inhibiting neurosphere formation and cell proliferation in vitro [163]. Thus far, there has been only one phase I clinical trial using BMPs, in particular BMP4 (NCT02869243), for which no results have been published.

**Table 1:** New cancer treatment strategies based on TGF-β. Preclinical and clinical trials based on TGF-β signaling to improve the treatment against GBM and other solid tumors.

	Treatment Strategies	The Role of TGF-β	Mice Model; Cells	References
Preclinicaltrials	hAMSCs as drug delivery vehicle	Use of TGF-β enhances homing capacity of hAMSCs	Male athymic nude mice; GBM086-Td-tomato *n* = 4 per group	[148]
	HSC gene therapy	TGF-β-blocking HSC gene therapy in combination with irradiation reduces tumor burden and prolongs survival	Female C57Bl/6J mice; GL261/Fluc or CT-2A/Fluc cells (1 × 10^5^) *n* = 11–16 per group	[149]
	NEO212	Blocks TGF-β and Notch pathways, arrests EndMT in vivo	Female athymic nude mice; 10^5^ USC02 and 3.3 × 10^4^ BEC cells *n* = 4 per group	[150]
	Cholesterol-lowering statins as adjunct therapy, simvastatin	Prolongs survival, inhibit TGF-β signaling	Female BALB/c SCID NCr; 500 G34 cells *n* = 5 per group	[151]
	Dual inhibition of α_v_β_3_ integrin and TβRI in GBM microenvironment as an adjuvant therapeutic target	Suppresses endothelial cell proliferation and M2 macrophage polarization	In vitro; RAW264.7 cells, GL261, CT-2A, ECs (C166-GFP)	[114]
	Inhibitors of the αv integrin/TGF-β axis in GSCs combined a with NK cell immunotherapy	NK cells conserve their cytotoxic function; αv integrin/TGF-β signaling in GSCs is blocked, as this axis is responsible for immune evasion	NSG human xenograft model; 0.5 × 10^6^ patient-derived GSC20 or GSC272 *n* = 4–5 per group	[157]
	DSF	Sensitizes resistant GBM to galunisertib (drug targeting TGF-β receptors)	NOD-SCID mice; radiation-resistant U87MG 2GR4, or radiation-resistant 1306 MG 3.5 GR6, or radiation–TMZ-resistant 1306 MG R6T3 (5 × 10^5^ cells) *n* = 4 per group	[158]
	oHSV with TβR inhibitor	Reinforces the antitumor effects of single intratumoral oHSV injections	Female SCID mice; recurrent MGG31 GSC (1 × 10^5^ cells) *n* = 6 per group	[159]
Clinical trials	Engineered natural killer (NK) cells *TGFBR2*-KO			NCT04991870
	AP12009 (TGF-β2 inhibitor) combined with temozolomide or PCV			NCT00431561
	AP12009 (TGF-β2 inhibitor) combined with temozolomide and drug delivery system			NCT00761280
	LY2157299 (TβRI inhibitor) plus lomustine			NCT01582269
	LY2157299 (TβRI inhibitor) plus radiation and temozolomide	The rate of disease control was higher in treatment with galunisertib plus radiotherapy compared to radiotherapy alone		NCT01220271 [160]
	Fresolimumab, neutralizing antibody	Phase 2 clinical trial No clinical benefit		NCT01472731 [131]
	M7824 bifunctional fusion protein targeting TGF-β and PD-L1	Good disease control in IDH-mutant GBM Phase 1 clinical trial		[161] NCT02517398

## 7. Conclusions

In conclusion, members of the TGF-β family are key players in glioblastoma progression. On the one hand, TGF-β1 and TGF-β2 promote tumorigenesis by inducing CSC self-renewal, tumor cell migration, and by repressing the immune system. On the other hand, BMPs induce CSC differentiation and block proliferation. Interestingly, BMPs can also promote tumor cell migration, and their role in the brain tumor immune response is not well studied. The signaling pathways of TGF-β and BMP are well studied, but the effectiveness of their use as either therapeutic targets or agents respectively has not been as good as expected, probably due to their complex signaling pathways and their ability to modulate not only cancer cells, but also the microenvironment. The combination of TGF-β inhibitors or recombinant BMP, together with other therapies such as TMZ or immunotherapy, are expected to have better efficacy in the treatment of glioblastoma.
